# The celiac ganglia and trunk: an assessment of anatomical variants and their clinical relevance

**DOI:** 10.25122/jml-2025-0015

**Published:** 2025-01

**Authors:** Diana-Theodora Morgos, Lucian-George Eftimie, Horia Nicolae, Remus Iulian Nica, Constantin Stefani, Daniela Miricescu, Adrian Tulin, Florin Mihail Filipoiu

**Affiliations:** 1 Doctoral School, Discipline of Anatomy, Carol Davila University of Medicine and Pharmacy, Bucharest, Romania; 2 Discipline of Anatomy and Biomechanics, Faculty of Physical Therapy, National University of Physical Education and Sports, Bucharest, Romania; 3 Dr. Carol Davila Central Military Emergency University Hospital, Bucharest, Romania; 4 Discipline of Neurology, Faculty of Medicine, Carol Davila University of Medicine and Pharmacy, Bucharest, Romania; 5 Discipline of Neurology, Elias University Emergency Hospital, Bucharest, Romania; 6 Discipline of General Surgery, Faculty of Medicine, Carol Davila University of Medicine and Pharmacy, Bucharest, Romania; 7 Surgery Department, Dr. Carol Davila Central Military Emergency University Hospital, Bucharest, Romania; 8 Department I of Family Medicine and Clinical Base, Dr. Carol Davila Central Military Emergency University Hospital, Romania; 9 Discipline of Biochemistry, Faculty of Dentistry, Carol Davila University of Medicine and Pharmacy, Bucharest, Romania; 10 Discipline of Anatomy, Carol Davila University of Medicine and Pharmacy, Bucharest, Romania

**Keywords:** clinical relevance, computed tomography angiography (CTA), anatomical variants, celiac ganglia, celiac trunk

## Abstract

The celiac ganglia are a network of nerve fibers that regulate various functions related to digestion, while the celiac trunk is a major artery that supplies oxygenated blood to the stomach, small intestine, and other organs in the upper abdominal region. Anatomical variants of these structures are common and can have significant implications for surgical and medical procedures. This prospective observational study was conducted over one year and included 300 patients (aged 45-75 years) with a history of peripheral arterial disease, evaluated at Dr. Carol Davila Central Military Emergency Hospital Bucharest, Romania, using a Philips Spectral CT 7500. The study identified three major anatomical variants of the celiac trunk, each associated with different positional distributions of the celiac ganglia. In cases where the celiac trunk presented as a hepatosplenic trunk with the left gastric artery originating from the abdominal aorta, the lateral position of the celiac ganglia was most prevalent. When the hepatosplenic trunk included the left gastric artery arising from the splenic artery, the postero-lateral position of the celiac ganglia was the most frequently observed. In patients with a hepatogastric trunk, where the splenic artery originated from the superior mesenteric artery, the lateral position of the celiac ganglia was again the most common. Statistical analysis reveals a t-statistic of 7.391 and 11.319 with a P value of 0.002. This article reviewed the anatomical variants of the celiac ganglia and their anatomical variants, highlighting their prevalence, clinical significance, and implications for surgical and interventional procedures.

## INTRODUCTION

The celiac artery, also known as the celiac axis or celiac trunk, is the initial significant branch of the abdominal aorta, arising from the ventral surface of the aorta just after it passes through the diaphragmatic aortic hiatus at the levels of the 12^th^ thoracic and 1^st^ lumbar vertebrae [1]. It typically trifurcates into the common hepatic artery, the left gastric artery, and the splenic artery, a configuration first reported by von Haller and referred to as 'tripus Halleri' [2]. This trunk supplies blood to the stomach, the duodenum, the liver, the gallbladder, the pancreas, and the spleen [3]. Two types of trifurcation patterns have been identified: a 'true' tripod occurs when all three arteries originate from a common trunk, forming a hepatogastrosplenic trunk, while a 'false' tripod describes a situation where one artery branches off before the others from the celiac trunk [4].

The variants of the celiac artery can influence the localization of the celiac ganglia by altering the vascular anatomy in the region, which may affect the nerve pathways and their spatial relationships [5]. For instance, if the celiac artery has atypical branching patterns, it could lead to variations in the positioning of the celiac plexus and ganglia, potentially shifting them closer to or further away from surrounding structures like the pancreas, kidneys, or abdominal aorta [6]. This relationship is crucial during surgical procedures, as understanding these variants may help surgeons avoid nerve injury and improve outcomes in procedures involving the abdominal region. These anatomical variations may also impact the distribution of pain or dysfunction experienced in conditions affecting the abdominal organs, warranting further investigation to enhance diagnostic and therapeutic strategies.

The celiac ganglia, also known as the celiac plexus, are a network of nerve fibers located in the retroperitoneal space. They are part of the autonomic nervous system and contribute to the modulation of pain perception. The celiac ganglia, in particular, play a crucial role in the innervation of the upper abdomen, and variants in their anatomy can lead to complex clinical presentations [7]. Despite their importance, anatomical variants of the celiac trunk and ganglia remain poorly understood and underreported. This scarcity of knowledge is partly because these variants are often discovered incidentally during surgery or autopsy rather than being actively sought out through systematic study [8].

This article aimed to shed light on the anatomical variants of the celiac ganglia and trunk, highlighting their prevalence, clinical significance, and implications for surgical and interventional procedures. By exploring the breadth of these variants, we hope to contribute to a deeper understanding of this complex region and improve patient care for individuals affected by various conditions.

## MATERIAL AND METHODS

### Study design

This cross-sectional cohort study was conducted at Dr. Carol Davila Central Military Emergency Hospital, Bucharest, Romania, between May 2023 and June 2024. Our cohort comprised 300 patients (160 men and 140 women) aged 45 to 70 years who underwent computed tomography angiography (CTA) for peripheral arterial disease (PAD) before surgery. Imaging was performed using a Philips Spectral CT 7500, an advanced spectral imaging system that enhances tissue characterization and improves diagnostic accuracy. Anatomical variations in the celiac trunk and ganglia are largely determined by genetic factors and embryological development rather than body composition. Therefore, age and gender were selected as the key demographic variables for this study (Table 1).

**Table 1:** Demographic characteristics of the study population

Characteristic	Value
*n*	300
Men	160
Women	140
Age range (years)	45-70
Median age	57.5

### Inclusion and exclusion criteria

Patients were considered eligible if they were diagnosed with peripheral arterial disease and were evaluated for surgical treatment—balloon angioplasty or stenting. Preoperative evaluation requires CTA before major vascular surgery as a standard indication, and the creatinine clearance, as an indicator of renal function, was required to meet a normal minimum threshold established at 90 mL/min.

Patients had to be at least 18 years old to be enrolled, and signed informed consent was mandatory for study participation. Patients were excluded in case of ongoing pregnancy or any associated medical history that would interfere with the study, for example, abdominal cancer.

### Data sources

For this study, the CTA of the celiac trunk was selected due to its essential role in identifying anatomical variants and aiding in clinical assessment. All patients underwent a non-contrast computer tomography (CT) scan and a contrast CT scan with arterial, portal, and late venous phases. Each patient was administered 1.5 ml/kg of a nonionic iodinated contrast agent (350 mg/mL) via a single-phase injection using an automated injector. The contrast agent was delivered at a uniform flow rate of 3 mL/s across all patients. The study protocol involved a baseline evaluation consisting of visualization of pre-operative abdominal CTA images and observation of the anatomical variant of the celiac trunk, which was consequently recorded in an Excel sheet. Immediately after that, the celiac ganglia localization was noticed and written down. After the data was completed, we performed statistical analysis to observe any potential correlation between the anatomical variant of the celiac trunk and celiac ganglia. Several factors, including image quality, patient anatomy, and pathologies, can influence the visualization of the celiac trunk and its branches in CT scans. However, in this study, all relevant celiac trunk branches were successfully visualized in every scan. There were no motion artifacts, inadequate contrast enhancement, or technical limitations during the scan acquisition. The acquired images underwent preprocessing to enhance quality by reducing noise and adjusting brightness and contrast for proper orientation. Anatomical landmarks were annotated, including the dimensions of the celiac ganglia and their relationships to adjacent structures, followed by measurements of distances to other landmarks.

### Outcome measures

The primary outcome measure was the analysis of CTA images to identify anatomical variants of the celiac trunk and celiac ganglia and investigate potential anatomical correlations between them. This study aimed to contribute to a deeper understanding of the anatomical variants of the celiac trunk and celiac ganglia, highlighting their clinical significance and implications for surgical and interventional procedures, such as celiac plexus block.

### Statistical analysis

Statistical analysis was performed using SPSS 26.0 for Windows (SPSS Inc., Chicago, IL, USA) and Microsoft Excel 2019. Data distribution was assessed using the Anderson-Darling and Shapiro-Wilk normality tests. A Bayesian one-sample *t*-test was applied to determine the statistical significance (*P* value) between two variables. The Pearson correlation test was used to evaluate the strength and direction of associations between continuous variables. An independent samples *t*-test was performed to compare means between two independent groups with normally distributed data. An ANOVA test was also used to assess whether observed group variations resulted from population differences or sampling variability. Finally, we used a regression analysis to examine the relationship between the dependent and independent variables, predict outcomes, and assess the strength and nature of those relationships.

## RESULTS

**Figure 1:**
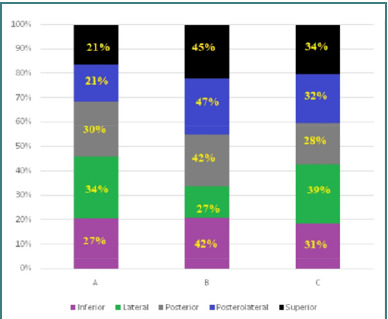
Distribution of anatomical variants of celiac ganglia in relation to the celiac trunk

The celiac trunk is a major arterial vessel that branches from the abdominal aorta, typically originating at the T12-L1 vertebral level. On CT imaging, it is visualized as a short, thick vessel branching into three primary arteries: the left gastric artery, the common hepatic artery, and the splenic artery [9]. The celiac ganglion is usually located just anterior to the celiac trunk, near the aorta, and may appear as a small, rounded structure in the retroperitoneum, often at the level of the first lumbar vertebra. Its most common locations include the anterior aspect of the aorta, the junction of the celiac trunk, and around the origin of the celiac trunk [10]. In this study, three anatomical variants of the celiac trunk were identified: Variant A – hepatosplenic trunk with the left gastric artery originating from the abdominal aorta; Variant B – hepatosplenic trunk with the left gastric artery originating from the splenic artery, and Variant C – hepatogastric trunk with the splenic artery originating from the superior mesenteric artery. Among the 300 patients, 90 had Variant A, 125 had Variant B, and 85 had Variant C. The anatomical variant of celiac ganglia concerning the celiac trunk was either inferior, lateral, posterior, postero-lateral, or superior. Figure 1 shows the distribution of anatomical variants of celiac ganglia concerning the celiac trunk.

**Figure 2:**
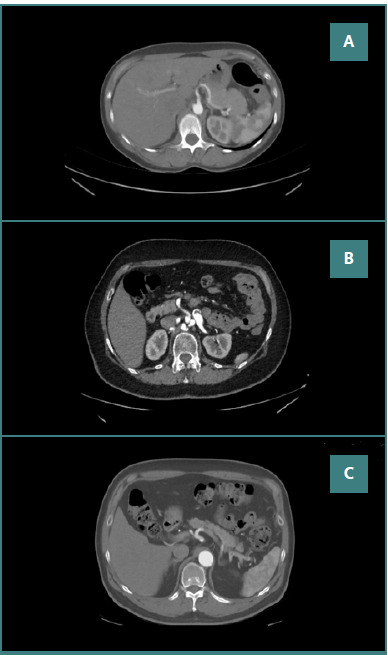
CTA imaging of celiac ganglia positions in relation to celiac trunk variants. A, Hepatogastric trunk, splenic artery from superior mesenteric artery, the celiac ganglion is posterolateral to the celiac trunk; B, Hepatosplenic trunk, left gastric artery from abdominal aorta, the celiac ganglion is posterior to the celiac trunk; C, Hepatosplenic trunk, left gastric artery from splenic artery, the celiac ganglion is inferior to celiac trunk.

The celiac ganglia were inferiorly positioned in 81 patients (27%) with Variant A (hepatosplenic trunk, left gastric artery from abdominal aorta), 126 patients (42%) with Variant B (hepatosplenic trunk, left gastric artery from splenic artery), and 93 patients (31%) with Variant C (hepatogastric trunk, splenic artery from superior mesenteric artery). The lateral position was observed in 102 patients (34%) with Variant A, 81 patients (27%) with Variant B, and 117 patients (39%) with Variant C. The posterior position was observed in 90 patients (30%) with Variant A, 126 patients (42%) with Variant B, and 84 patients (28%) with Variant C. The postero-lateral position was observed in 63 patients (21%) with Variant A, 141 patients (47%) with Variant B, and 96 patients (32%) with Variant C. The superior position was observed in 63 patients (21%) with Variant A, 135 patients (45%) with Variant B, and 102 patients (34%) with Variant C. In Variant A, the ganglia were most frequently lateral to the celiac trunk, whereas in Variant B, the postero-lateral position was dominant. In Variant C, the lateral position was again the most prevalent. Figure 2A-C shows CTA-acquired images of the anatomical variant of celiac ganglia in relation to the celiac trunk.

Hepatogastric trunk, splenic artery from superior mesenteric artery, the celiac ganglion is posterolateral to the celiac trunk; B, Hepatosplenic trunk, left gastric artery from abdominal aorta, the celiac ganglion is posterior to the celiac trunk; C, Hepatosplenic trunk, left gastric artery from splenic artery, the celiac ganglion is inferior to celiac trunk.

No significant differences were observed in the distribution of celiac trunk variants and celiac ganglia positions across age and gender groups. Table 2 summarizes the posterior distribution characteristics of the pairwise correlations between celiac ganglia positioning relative to the celiac trunk and the anatomical variant of the celiac trunk.

**Table 2:** Posterior distribution characterization of pairwise correlations between celiac ganglia position and celiac trunk variants

Variable			Celiac ganglia position relative to celiac trunk	Anatomical variant of celiac trunk
Celiac ganglia position relative to celiac trunk	Posterior	Mode	N/A	-0.128
		Mean	N/A	-0.092
		Variance	N/A	0.084
	95% Credible interval	Lower Bound	N/A	-0.635
		Upper Bound	N/A	0.453
	*n*		5	3
Anatomical variant celiac trunk	Posterior	Mode	-0.128	N/A
		Mean	-0.092	N/A
		Variance	0.084	N/A
	95% Credible interval	Lower Bound	-0.635	N/A
		Upper Bound	0.453	N/A
	*n*		5	3

The analysis assume reference priors (c = 0)

The posterior distribution of the correlation coefficient was estimated using Bayesian inference. The posterior mode was -0.128, representing the most frequently occurring value in the posterior distribution. The mean correlation was -0.092, indicating the average value of the posterior distribution, with a variance of 0.084, which quantifies the variability of the posterior distribution. The analysis employed a non-informative prior, indicated by c = 0. The posterior distribution implies that the correlation between the two variables was likely around -0.09, with the credible interval suggesting a 95% probability that the true correlation ranged between -0.63 and 0.45. Table 3 presents the results of a Bayesian one-sample *t*-test for two variables: "celiac ganglia related to the anatomical variant of the celiac trunk" and "anatomical variant celiac trunk".

**Table 3:** Bayesian Factor for one-sample *t*-test for celiac ganglia position and celiac trunk variants

	Celiac ganglia position relative to the celiac trunk	Anatomical variant of the celiac trunk
*n*	5	3
Mean	60.00	60.00
Std. Deviation	8.276	8.602
Std. Error Mean	8.118	5.301
Bayes Factor^a^	0.032	0.009
t	7.391	11.319
df	4	4
Sig.(2-tailed)	0.002	<0.001

The Bayesian analysis assessed whether the sample means of specific anatomical variants related to the celiac trunk significantly differed from a hypothetical population mean of zero. The reference population included various anatomical variants of the celiac trunk, such as classical branching patterns versus atypical branches, with proportions reflecting clinical relevance and anatomical variability typically seen in the literature. The analysis yielded a sample mean of 60.00 for both variables. The standard deviations were 8.276 and 8.602, indicating moderate variability within the data. The standard error of the mean (SEM) for the first variable was 8.118, and for the second, 5.301, suggesting more precision in estimating the population mean for the second variable. The computed Bayes Factors (BF) were 0.032 and 0.009, both of which were less than 1, indicating that the evidence favors the null hypothesis (H0: μ = 0) over the alternative hypothesis (H1: μ ≠ 0) but not conclusively since a lower BF indicates greater support for the null hypothesis but does not negate the presence of significance. The t-statistic values were 7.391 and 11.319, with four degrees of freedom for both variables. The *P* values (Sig.) were 0.002 and <0.001, respectively, which indicates that the sample means were significantly different from 0 at a significance level of 0.05.

The results suggest a significant mean difference from 0 for both variables, providing strong evidence against the null hypothesis. In the context of anatomical variants of celiac ganglia related to the celiac trunk, this result suggests that the mean value of celiac ganglia related to the anatomical variant of the celiac trunk was significantly different from 0, indicating that there was a systematic difference in the distribution of celiac ganglia between individuals with an anatomical variant of the celiac trunk. The Bayes Factor (0.032) suggests the data provide strong evidence for this finding. These results suggest that individuals with an anatomical variant of the celiac trunk tend to have a different distribution of celiac ganglia compared to those without this variant. This could have implications for understanding the anatomy and function of the celiac trunk and its relationship to the surrounding neural structures. Table 4 presents the results of the ANOVA test based on the data already determined.

**Table 4:** ANOVA test

Model	df	F	*P*
Regression	4	1.6	0.002

The ANOVA results indicate that the regression model shows the effect of a predictor (independent variable) on a response (dependent variable). The model had four degrees of freedom (df = 4), and the F-value of 1.6 suggests that the explained variance was moderate relative to the total variance in the dataset. The *P* value of 0.02 was less than the level of 0.05, indicating a statistically significant relationship between the independent and dependent variables. In this context, it suggests that the independent variable significantly explained variations in the dependent variable. The independent variable had a significant effect on the dependent variable. Table 5 displays the regression analysis results conducted based on the previously gathered data.

**Table 5:** Regression analysis of anatomical variants of celiac ganglia related to celiac trunk

Predictor variable	Estimate	Std. Error	t value	*P* value
Intercept	-0.57	0.32	-1.79	0.076
Celiac_ganglia (Inferior)	0.41	0.24	1.73	0.089
Celiac_ganglia (Lateral)	-0.23	0.21	-1.09	0.028
Celiac_ganglia (Posterior)	-0.47	0.26	-1.83	0.041
Celiac_ganglia Postero-lateral)	0.31	0.23	1.34	0.019
Celiac_ganglia (Superior)	-1.01	0.36	-2.83	0.006

The analysis presents regression results assessing the influence of five different celiac ganglia anatomical variant variables on an outcome, the anatomical variants of the celiac trunk, with varying significance levels, in the general population. The intercept is negative and marginally significant (*P* = 0.076), indicating a potential baseline effect. Among the celiac ganglia anatomical variants variables, the latter three anatomical variants (posterior, posterolateral, superior) showed a strong negative influence with a statistically significant *P* value. Conversely, the first two celiac ganglia anatomical variants (inferior, lateral) had weaker and non-significant effects.

## DISCUSSION

The clinical significance of anatomical variants in the celiac trunk and celiac ganglia is of considerable interest, particularly in surgical and interventional procedures such as celiac plexus block [11]. Surgical procedures involving the upper gastrointestinal tract, such as pancreaticoduodenectomy (Whipple procedure), distal gastrectomy, and laparoscopic cholecystectomy, require a thorough understanding of the anatomical relationships between the celiac trunk and ganglia [12]. Variations in these structures can affect upper abdominal surgeries [13] and may increase the risk of surgical complications [14].

The celiac trunk and ganglia have notable anatomical variants that can significantly influence clinical practice [15]. These differences are crucial for understanding the vascular anatomy of individuals, as they may impact essential structures such as the liver, the pancreas, the spleen, and the duodenum [15]. Aberrant ganglia may complicate surgical procedures if misidentified, potentially leading to inadvertent injury to surrounding tissues and increased risk of complications such as chronic pain or digestive dysfunction [16]. An in-depth understanding of the anatomical relationships involving the celiac trunk and ganglia is vital for the success of upper gastrointestinal surgeries and interventional procedures. Awareness of potential vascular variations allows surgeons to modify their techniques to minimize the risk of complications, such as hemorrhage, organ ischemia, and inadvertent damage to neural structures. Tailoring surgical dissection strategies based on anatomical findings can improve outcomes, including reduced operative times and lower complications. Recognizing the specific characteristics of the celiac trunk and ganglia is fundamental in enhancing patient safety and preserving vital organ function [17].

Advanced imaging techniques, particularly CT, play a critical role in preoperative planning for surgeries involving the celiac trunk and ganglia. CT angiography provides detailed, non-invasive visualization of vascular structures, helping to identify anatomical variants that could influence surgical outcomes. Multi-slice CT, in particular, allows for a comprehensive evaluation of the branching patterns of the celiac trunk and the precise location of the celiac plexus. This imaging capability enables surgeons to plan their approach, mitigate risks, and enhance intraoperative decision-making through three-dimensional reconstructions [18]. A thorough preoperative assessment of these variants using imaging modalities such as CT or magnetic resonance imaging (MRI) can help inform surgical planning and minimize the risk of complications [19]. Imaging studies play a crucial role in identifying and characterizing anatomical variants of the celiac ganglia and the celiac trunk. These investigations provide important insights for surgical planning, diagnosis of related pathologies, and understanding the variants in vascular anatomy. The following imaging modalities are commonly used: CT, CT angiography, and MRI.

CT angiography is a non-invasive technique that provides detailed images of the blood vessels. They are particularly useful for assessing the celiac trunk and its branches, as well as visualizing the anatomical position and configuration of the celiac ganglia. MRI is not as commonly used as CT for vascular structures but can provide detailed images of soft tissue and may be useful in certain situations to evaluate the relationship between the celiac trunk, ganglia, and adjacent structures. It is well established that healthy individuals typically do not undergo angiographic CT scans solely to investigate anatomical details. Additionally, the intrinsic anatomy of structures such as the celiac ganglia remains unchanged in individuals with peripheral arterial disease, as this condition does not alter congenital anatomical features. Therefore, although this study's findings are based on a cohort with PAD, they remain applicable to the general population, including individuals without vascular pathology. This highlights the continuity of fundamental anatomical structures regardless of pathological states.

Doppler ultrasound can visualize blood flow in the celiac trunk and its branches but is limited in its ability to detail anatomical variants [20]. It may be useful in younger patients or those who require a non-radiative assessment. Endovascular techniques are another kind of imaging study, such as catheter angiography, that may be applied, especially if there is a need to assess vascular anatomy in the context of surgery or interventional procedures [21]. Imaging studies play a crucial role in identifying anatomical variants, including variations in the branching pattern of the celiac trunk and abnormal positions or sizes of the celiac ganglia. This knowledge is essential for surgical planning, as anatomical differences may require modifications to standard surgical approaches in upper abdomen procedures. Additionally, imaging studies contribute to research on anatomical diversity, providing essential insights that enhance the understanding of the celiac trunk and ganglia’s complex anatomy. This knowledge is critical for improving medical education and surgical training, ensuring that healthcare professionals are well-equipped to navigate anatomical variations in clinical practice [22,23].

The utilization of local anesthetics or neurolytic agents in celiac plexus block helps interrupt pain pathways, improving the quality of life for patients. This procedure is primarily used for pain management, particularly in cases of severe abdominal pain that are unresponsive to conventional treatments. This intervention provides significant symptom relief by blocking nerve signals transmitted through the celiac plexus, a network of nerves near the aorta in the upper abdomen. The procedure requires anatomical knowledge of celiac ganglia and their variations concerning the anatomical variants of the celiac trunk to avoid vascular structures and accurately locate the celiac ganglia [24]. The procedure is usually performed under imaging guidance, such as fluoroscopy or ultrasound, with local anesthetic injection or sedation. It has many benefits, including reducing opioid use and its associated side effects. While generally safe, potential complications can include infection, bleeding, and damage to surrounding structures, which is the main reason to recognize the significance of well-known local anatomy, including anatomical variants. Patients are often monitored post-procedure to assess pain relief and side effects. Follow-up appointments may be scheduled to evaluate ongoing pain management needs [25]. A celiac plexus block can be an effective procedure for managing intractable abdominal pain, offering significant relief for individuals with gastrointestinal conditions, for example, abdominal cancer.

As for considering the limitations of the study, we mention that recruiting all participants from a single center with the same medical history of peripheral arterial disease can introduce bias into the study results, as it may not accurately reflect the anatomical variations present in the broader population. Nonetheless, the information gleaned from a single center can still provide valuable insights, particularly for clinicians managing similar conditions. It is essential to acknowledge that patients with diseases like peripheral arterial disease tend to undergo angiographic CT imaging due to specific symptomatic presentations, making their anatomical features pertinent for surgical planning and intervention in affected populations while emphasizing that the results primarily serve applied clinical contexts rather than broad anatomical norms.

## CONCLUSION

This study examined the anatomical variants of the celiac trunk and the corresponding distributions of celiac ganglia in a cohort of 300 patients, revealing distinct patterns associated with three variants of the celiac trunk. Notably, the hepatosplenic trunk and left gastric artery from the splenic artery (Variant B) had the highest variety of celiac ganglia anatomical variants, showing significant frequencies of inferior and postero-lateral ganglia locations. The statistical analysis indicated that individuals with anatomical variations of the celiac trunk had systematic differences in celiac ganglia distribution, highlighting their implications in celiac plexus block and preoperative care.
